# Prehospital Lactated Ringer's Solution Treatment and Survival in Out-of-Hospital Cardiac Arrest: A Prospective Cohort Analysis

**DOI:** 10.1371/journal.pmed.1001394

**Published:** 2013-02-19

**Authors:** Akihito Hagihara, Manabu Hasegawa, Takeru Abe, Yoshifumi Wakata, Takashi Nagata, Yoshihiro Nabeshima

**Affiliations:** 1 Kyushu University Graduate School of Medicine, Department of Health Services Management and Policy, Fukuoka, Japan; 2 Ambulance Service Planning Division, Fire and Disaster Management Agency, Ministry of Internal Affairs and Communications, Tokyo, Japan; 3 Kyushu University Hospital, Department of Emergency and Critical Care Center, Fukuoka, Japan; Free University of Brussels, Belgium

## Abstract

**Background:**

No studies have evaluated whether administering intravenous lactated Ringer's (LR) solution to patients with out-of-hospital cardiac arrest (OHCA) improves their outcomes, to our knowledge. Therefore, we examined the association between prehospital use of LR solution and patients' return of spontaneous circulation (ROSC), 1-month survival, and neurological or physical outcomes at 1 month after the event.

**Methods and Findings:**

We conducted a prospective, non-randomized, observational study using national data of all patients with OHCA from 2005 through 2009 in Japan. We performed a propensity analysis and examined the association between prehospital use of LR solution and short- and long-term survival. The study patients were ≥18 years of age, had an OHCA before arrival of EMS personnel, were treated by EMS personnel, and were then transported to hospitals. A total of 531,854 patients with OHCA met the inclusion criteria. Among propensity-matched patients, compared with those who did not receive pre-hospital intravenous fluids, prehospital use of LR solution was associated with an increased likelihood of ROSC before hospital arrival (odds ratio [OR] adjusted for all covariates [95% CI] = 1.239 [1.146–1.339] [*p*<0.001], but with a reduced likelihood of 1-month survival with minimal neurological or physical impairment (cerebral performance category 1 or 2, OR adjusted for all covariates [95% CI] = 0.764 [0.589–0.992] [*p* = 0.04]; and overall performance category 1 or 2, OR adjusted for all covariates [95% CI] = 0.746 [0.573–0.971] [*p* = 0.03]). There was no association between prehospital use of LR solution and 1-month survival (OR adjusted for all covariates [95% CI] = 0.960 [0.854–1.078]).

**Conclusion:**

In Japanese patients experiencing OHCA, the prehospital use of LR solution was independently associated with a decreased likelihood of a good functional outcome 1 month after the event, but with an increased likelihood of ROSC before hospital arrival. Prehospital use of LR solution was not associated with 1-month survival. Further study is necessary to verify these findings.

*Please see later in the article for the Editors' Summary*

## Abstract 2

In a cohort of more than 500,000 individuals who experienced out-of-hospital cardiac arrest in Japan, Akihito Hagihara and colleagues studied whether administration of lactated Ringer's solution was associated with survival and functional outcomes.

## Editors' Summary

**Background:**

Cardiac arrest, a condition in which the heart suddenly stops pumping, is caused by problems with the heart's internal electrical system, which controls the rate and rhythm of the heart contractions that pump blood around the body. If this electrical system malfunctions, an abnormal heartbeat or “arrhythmia” develops that, in some cases, causes cardiac arrest. Because blood is no longer being pumped around the body, the organs and tissues of the body do not receive the oxygen they need to function. Consciousness is lost immediately and, if medical attention is not provided quickly, death follows within a few minutes—about 95% of people who have a cardiac arrest die before they reach hospital or emergency medical help. Moreover, survivors of cardiac arrest are often left with permanent damage to the brain and other organs. Early cardiopulmonary resuscitation (CPR; chest compression to pump the heart and mouth-to-mouth resuscitation to inflate the lungs) and early defibrillation (delivery of an electric shock to the heart to restore its normal rhythm) reduce the risk of death and permanent organ damage after cardiac arrest.

**Why Was This Study Done?:**

Another procedure that is sometimes used during pre-hospital resuscitation of cardiac arrest cases is intravenous fluid administration—delivering liquid into a vein through an intravenous needle. A solution that is often used for this purpose is lactated Ringer's (LR) solution, a mixture of inorganic salts and sodium lactate. However, the effects of intravenous LR solution on the outcomes of patients who have an out-of-hospital cardiac arrest have not been studied. In this prospective cohort analysis, the researchers examine the association between the pre-hospital use of LR solution and the return of spontaneous circulation, one-month survival, and neurological and physical outcomes at one month after cardiac arrest among patients in Japan who have had an out-of-hospital cardiac arrest. A prospective cohort analysis identifies a group of patients with a specific condition and examines how they subsequently fare.

**What Did the Researchers Do and Find?:**

In Japan, the Fire and Disaster Management Agency records all out-of-hospital cardiac arrest cases in a nationwide database. The researchers used this database to identify more than half a million out-of-hospital cardiac arrest cases that occurred in Japan between 2005 and 2009. To examine the association between pre-hospital use of LR solution and short- and long-term survival, the researchers used a statistical technique called propensity analysis. This technique is used in observational studies to control for confounding—unknown differences between people who receive an intervention and those who do not receive an intervention that might affect outcomes and thus make it hard to draw conclusions about the intervention's true effects. By examining a large number of variables (for example, age, sex, and time taken for help to arrive), the researchers gave every patient a propensity score that indicated their probability of receiving pre-hospital LR solution, and then used this score to match each patient who received LR solution with a similar patient who did not receive LR solution. Among propensity-matched patients, pre-hospital use of LR solution was associated with a slightly increased chance of return of spontaneous circulation before arrival at a hospital and with a decreased chance of 1-month survival with minimal neurological or physical impairment. Among the whole cohort, pre-hospital use of LR solution was not associated with overall one-month survival.

**What Do These Findings Mean?:**

These findings suggest that, among Japanese patients with out-of-hospital cardiac arrest, pre-hospital use of LR solution was associated with less chance of good functional outcomes at one month. However, the present study has several limitations. For example, data on in-hospital treatment following out-of-hospital cardiac arrest were not available, so the outcome differences between patients receiving and not receiving LR solution potentially could reflect differences in their in-hospital treatment. Moreover, although the researchers undertook a propensity analysis, this study, like all observational studies, can only partly control for selection bias and confounding factors. Thus, the observed associations between LR solution use and short- and long-term outcomes may actually reflect the effects of some unknown characteristic shared by the patients who received LR solution. Because of these and other limitations, it is essential that the findings of this study are verified before recommendations about the pre-hospital use of LR solution in patients with out-of-hospital cardiac arrest are made.

**Additional Information:**

Please access these Web sites via the online version of this summary at http://dx.doi.org/10.1371/journal.pmed.1001394.

- The US National Heart Lung and Blood Institute provides information on sudden cardiac arrest and on heart arrhythmias
- The American Heart Association also information in several languages on sudden cardiac death and on arrhythmias; a selection of personal stories about arrhythmia and cardiac arrest is also available
- The not-for-profit Sudden Cardiac Arrest Foundation provides information on all aspects of cardiac arrest, including survivor stories
- MedlinePlus provides links to other resources about cardiac arrest (in English and Spanish)

## Introduction

Intravenous (IV) fluid loading is performed during prehospital resuscitation for patients who have out-of-hospital cardiac arrest (OHCA) in Japan. Lactated Ringer's (LR) solution is commonly used as an intravenous fluid [1]–[3], and in Japan, a ministerial ordinance mandates that LR solution be used during prehospital intravenous fluid loading [2],[3]. However, whether administering LR solution to patients with OHCA is beneficial is unknown. Cardiac arrest is a primary cause of lactic acidosis, and prehospital intravenous LR solution loading may worsen this acidosis [4]. According to our systematic literature review on arterial lactate concentrations among patients with OHCA, these patients have already developed lactic acidosis at hospital admission in the majority of studies (Table S1). Specifically, 20 of 24 studies reported that arterial lactate concentration at hospital admission was equal to or higher than the lactic acidosis criterion value (i.e., 5–6 mmol/l) (Table S1) [5]. In the remaining four studies, although mean lactate concentration was lower than the criterion value, these studies included only patients with OHCA who regained spontaneous circulation before hospital arrival, and who had other attributes such as witnessed collapse, shockable rhythm, and short no-flow time (Table S1, numbers 10, 11, 12, 17).

Additionally, there are two isomers of lactate (i.e., D-lactate, L-lactate) [6],[7]. L-lactate is part of normal human metabolism and does not activate neutrophils, whereas D-lactate is not part of normal human metabolism and accumulates and activates neutrophils [8]. Evidence indicates that LR solution with racemic lactate (i.e., an equal mixture of D(−)-isomers and L(+)-isomers) influences neutrophil function and leukocyte gene expression [6]. The process of activating neutrophils by D-lactate may be the trigger for acute respiratory distress syndrome [9],[10].

Thus far, the outcomes of routine IV fluid administration versus no fluid administration during cardiopulmonary resuscitation (CPR) in humans have not been directly compared. Most human and animal studies of fluid infusion during CPR did not have a control group [11],[12]. Two animal studies showed that normothermic fluid infusion during CPR caused a decrease in central pulmonary pressure [13],[14]. In addition to normothermic fluid, hypertonic and chilled fluids have been studied in animal studies and small studies in humans and have not shown a survival benefit [11],[12],[15]. Specifically, to our knowledge, no studies have evaluated the effects of using prehospital LR solution on outcomes of patients with OHCA. Therefore, we performed a propensity analysis using national data of all patients with OHCA from 2005 through 2009 in Japan and examined the association between the use of LR solution before hospital arrival vs no IV fluid and return of spontaneous circulation (ROSC), 1-month survival, and neurological or physical outcomes at 1 month after the event [16].

## Methods

### Ethics Statement

This was a prospective observational study using national registry data. This study was approved by the ethics committee at Kyushu University Graduate School of Medicine. The requirement for written informed consent was waived.

### Data Collection

The emergency medical service (EMS) system in Japan has been described elsewhere [17],[18]. Briefly, EMS was provided through 807 fire stations with municipal government dispatch centers, and a tiered response (119 for fire and ambulance). As the Japanese guidelines do not allow EMS providers to terminate resuscitation in the field, all patients with OHCA who are treated by EMS personnel are transported to hospitals, excluding those with decapitation, incineration, decomposition, rigor mortis, or dependent cyanosis [19]. An ambulance crew consists of three EMS personnel, including as least one emergency lifesaving technician. They carry a defibrillator on which monophasic or biphasic waveforms can be selected. Specially trained emergency lifesaving technicians have been permitted to insert an IV line with approval from an online emergency physician as prehospital emergency care since July 2004 [17],[20]. Since the Medical Practitioner's law prohibits medical treatments by individuals other than medical doctors in Japan [21], this online control is required nationwide. In addition to inserting an IV line, epinephrine administration and advanced airway management also require approval from an online emergency physician. The necessity of these advanced life support (ALS) measures is judged on the basis of a patient's condition. The Fire and Disaster Management Agency (FDMA) registers all OHCA cases in a prospective, nationwide, population-based database using the standardized Utstein-style template. These data are initially handwritten. Then, EMS personnel in cooperation with the physicians in charge of the patients with OHCA summarize each OHCA case in the standardized Utstein style [19],[22]. Data from the 807 fire stations with dispatch centers in the 47 prefectures are then integrated into a national registry system on the FDMA database server after an electronic data check by FDMA.

### Participants

The study patients were ≥18 y of age, had an OHCA before arrival of EMS personnel, were treated by EMS personnel, and were then transported to medical institutions between January 1, 2005, and December 31, 2009.

### Lactated Ringer's Solution

The Ministerial Ordinance of the Japanese Government mandates that LR solution be used in prehospital fluid resuscitation.[2],[3], and in the study sample, when IV fluid was administered before ROSC, LR solution was used in at least 91.2% of cases. Between 2006 and 2009, two types of LR solutions (L(+)-isomers and racemic) were used, and 2.3% of all medical facilities used the LR solution with racemic lactate [23]. Products in the L (+)-isomer group and the racemic group were isotonic (i.e., the ratio of osmotic pressure to physiological salt solution = 0.9–1.0), and their pH was 6.7 [23]. To the best of our knowledge, relevant data about the volume of prehospital LR solution administered is not available in Japan. However, based on our experience, the mean volume received by the intervention group during the mean transportation time (27 min) is estimated to have been between 200 and 300 ml. LR solution was kept at room temperature until it was used. The temperatures of the patients were not recorded in this study. However, according to a previous study of OHCA cases in Japan, in which the patient's collapse was witnessed and the time from call to hospital arrival was similar to that of the present study, the patients' temperatures were 34.5°C–34.8°C [24].

The origin of cardiac arrest (i.e., cardiac or noncardiac) was determined clinically by the physician in charge, with the aid of EMS personnel. Patients who were hypovolemic at the time of out-of-hospital arrest were classified as non-cardiac. The main five subcategories of non-cardiac arrest were treated with LR in the following percentages of cases: cerebrovascular diseases, 20.8% (*n* = 40,144); respiratory diseases, 16.8% (*n* = 32,424); malignant tumors, 13.1% (*n* = 25,283); trauma, 18.8% (*n* = 36,248); and others, 19.5% (*n* = 37,635).

### Study Variables

Study variables are listed by prehospital LR solution use status in Table 1. Because an automated external defibrillator (AED) analyzes a patient's rhythm automatically and delivers a shock only when it detects ventricular fibrillation (VF), the patient's first recorded rhythm was regarded as VF when laypersons delivered shocks with the use of a public-access AED. The VF category also included ventricular tachycardia (VT). Time between the call to the scene and hospital arrival was measured using dispatch records at the fire station and an emergency lifesaving technician's watch. Neurological outcomes 1 month after the event were evaluated using the five categories of the Cerebral Performance Category (CPC) scale (1, good cerebral performance; 2, moderate cerebral disability; 3, severe cerebral disability; 4, coma or vegetative state; 5, death). Physical status 1 month after the event was evaluated using the five categories of the Overall Performance Category (OPC) scale (1, no or mild neurological disability; 2, moderate neurological disability; 3, severe neurological disability; 4, coma or vegetative state; 5, death) [19],[22],[25]. The EMS person in charge of each patient with OHCA had a face-to-face meeting with the doctor who treated that patient at the hospital to collect these 1-month follow-up data. If the patient was no longer at the hospital, the EMS personnel conducted a follow-up search, and information on survival 1 month after the event was collected. If information on a patient's cognitive or physical function at his/her new address was not available, the latest available data were used.

**Table:** **Table 1.** Baseline characteristics of patients with OHCA according to LR solution use: 2005–2009 national data in Japan (*n = *531,854).

Variable	*n* (%) LR Use (*n = *109,140)	*n* (%) No LR Use (*n = *422,714)	*p*-Value
***(OHCA patients)***			
Cases by year			
2005	10,607 (9.72)	89,900 (21.27)	<0.001
2006	17,970 (16.47)	85,739 (20.29)	
2007	23,410 (21.45)	80,443 (19.03)	
2008	26,946 (24.69)	83,670 (19.80)	
2009	30,185 (27.66)	82,896 (19.61)	
Age (y) (SD)	72.50 (15.66)	72.70 (16.55)	<0.001
Sex (male)	67,579 (61.92)	244,939 (57.94)	<0.001
Bystander eyewitness (yes)	44,852 (41.10)	170,703 (40.38)	<0.001
Relationship between bystander and patient (family member)	26,506 (24.29)	85,213 (20.16)	<0.001
Origin of OHCA, cardiac origin	63,994 (58.63)	230,747 (54.59)	<0.001
***(CPR initiated by bystander)***			
Chest compressions (yes)	44,584 (41.49)	154,492 (36.81)	<0.001
Rescue breathing (yes)	15,998 (15.00)	60,190 (14.38)	<0.001
Use of public-access AED (yes)	14,999 (13.83)	42,705 (10.11)	<0.001
***(Life support by EMS personnel)***			
Emergency life-saving technician in ambulance (yes)	108,327 (99.26)	390,994 (92.50)	<0.001
Medical doctor in ambulance (yes)	3,850 (3.53)	9,616 (2.28)	<0.001
Advanced life support by MD (yes)	16,063 (14.72)	64,665 (15.31)	<0.001
Time from call to arrival at scene (min) (SD)	7.52 (3.92)	7.23 (3.79)	<0.001
Time from call to arrival at hospital (min) (SD)	36.10 (12.99)	31.13 (13.36)	<0.001
First documented rhythm			
VF/pulseless VT	10,261 (9.40)	29,506 (6.98)	<0.001
PEA/asystole	98,879 (90.60)	393,208 (93.02)	
Defibrillation by EMS personnel (yes)	14,999 (13.83)	42,705 (10.11)	<0.001
Use of ALS devices (laryngeal mask/an adjunct airway/tracheal tubes)	81,018 (74.23)	151,053 (35.73)	<0.001
Epinephrine use (yes)	25,104 (23.12)	1,040 (0.25)	<0.001
(***Endpoints)***			
ROSC before hospital arrival (ROSC) (yes)	9,589 (8.79)	25,172 (5.95)	<0.001
1-mo survival after cardiac arrest (yes)	4,839 (4.43)	21,166 (5.01)	<0.001
Cerebral performance category 1 mo after the event (good performance/moderate disability)	1,648 (1.51)	10,720 (2.54)	<0.001
Overall performance category 1 mo after the event (no or mild neurological disability/moderate neurological disability)	1,654 (1.52)	10,610 (2.51)	<0.001

ALS, advanced life support; PEA, pulseless electrical activity; SD, standard deviation; VT, ventricular tachycardia.

### Endpoints

Endpoints were ROSC before hospital arrival; survival at 1 month after cardiac arrest; survival at 1 month with minimal neurological impairment, defined as CPC category 1 or 2; and survival at 1 month with minimal neurological disability, defined as OPC category 1 or 2 (Table 1) [19],[22],[25].

### Statistical Analysis

**Figure 1:**
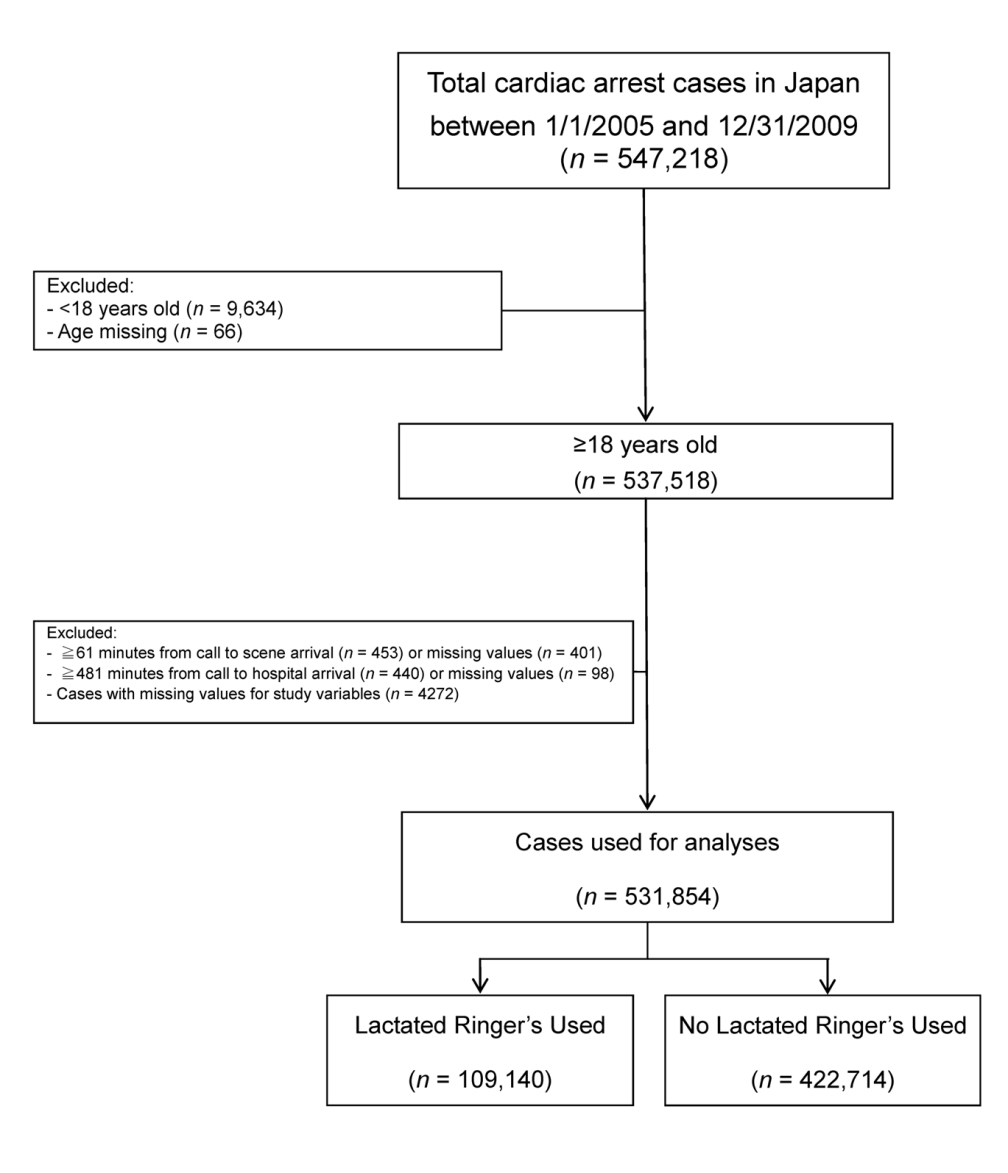
OHCA cases between 2005 and 2009 that were used for analyses.

Of the data of patients who had OHCAs between January 1, 2005, and December 31, 2009, in Japan and who were entered into the national registry (*n = *547,218), data that met the inclusion criteria concerning patient age and time course were analyzed (*n = *531,854) (Figure 1).

Prehospital LR solution use was not randomly assigned to the patient population. Thus, to control for potential confounding and selection biases, rather than using the propensity score as a covariate, we developed a propensity score for LR solution use before hospital arrival and used this for matching [10]. Specifically, in the first step, we used a full non-parsimonious logistic regression model with prehospital LR solution use as a dependent variable; this model included as independent variables every variable except for the three endpoint variables (i.e., 1-month survival and CPC and OPC scores) as shown in Table 1 (i.e., 67 variables including four dummy variables for “cases by year” and 46 dummy variables for the 47 prefectures in Japan). A propensity score for LR solution use before hospital arrival was calculated from the logistic regression equation for each patient. This propensity score represented the probability of prehospital LR solution use. In the second step, on the basis of the propensity score, patients who received prehospital LR solution were matched to unique control patients who did not receive prehospital LR solution [26]. The matching algorithm was greedy match, which is frequently used to match cases to controls in observational studies. Once a match is made, the match is not reconsidered.

Using data for all cases, three unconditional multiple logistic regression models were fit, using one of the endpoint variables in Table 1 as a dependent variable. Specifically, starting with (1) an unadjusted model, we adjusted for the effects of (2) variables shown to be factors in resuscitation outcome in previous studies; and (3) all study variables in Table 1. Using the propensity-matched patient data, conditional multiple logistic regression models were fitted using one of the endpoint variables in Table 2 as a dependent variable. Specifically, starting with (1) an unadjusted model, we adjusted for the effects of (2) propensity; (3) propensity and significant variables in the propensity-matched sample in Table 2; (4) propensity, significant variables in the propensity-matched sample in Table 2, and variables shown to be factors in resuscitation outcome in previous studies; and (5) propensity and all study variables in Table 1. In total, five models were fitted. With actual rates of ROSC, 1-mo survival, CPC category 1 or 2 after the event, OPC category 1 or 2 equal to 8.79%, 4.43%, 1.51%, and 1.52%, respectively, in the prehospital LR solution group, and rates of 5.95%, 5.01%, 2.54%, and 2.51%, respectively, in the prehospital no-LR solution group, among all patients (Table 1), 109,140 samples for each group provided the power levels summarized in Table 3 with a type I error of 5% or 1% [27]. With actual rates of ROSC, 1-mo survival, CPC category 1 or 2 after the event, OPC category 1 or 2 after the event rates of 6.29%, 4.25%, 1.59%, and 1.58%, respectively, in the prehospital LR solution group, and 5.22%, 4.07%, 1.79%, and 1.79%, respectively, in the prehospital no-LR solution group of propensity-matched individuals (Table 2), 76,293 samples for each group provided the power levels summarized in Table 3 with a type I error of 5% or 1% [27]. The two-sided significance level for all tests was *p*<0.05. All analyses were performed using SAS version 8.2 software (SAS Institute).

**Table 2:** Baseline characteristics of patients with OHCA according to LR solution use in propensity-matched patients with OHCA: 2005–2009 national data in Japan (*n = *152,586).

Variable	*n* (%) LR Use (*n* = 76,293)	*n* (%) No LR Use (*n* = 76,293)	*p-V*alue
***(OHCA patients)***			
Cases by year			
2005	9,987 (13.09)	9,819 (12.87)	0.002
2006	15,493 (20.31)	15,005 (19.67)	
2007	15,220 (19.95)	15,215 (19.94)	
2008	17,581 (23.04)	17,749 (23.26)	
2009	18,012 (23.61)	18,505 (24.26)	
Age (y) (SD)	72.52 (15.73)	72.44 (16.22)	0.37
Sex (male)	46,371 (60.78)	46,564 (61.03)	0.31
Bystander eyewitness (yes)	28,129 (36.87)	27,727 (36.34)	0.03
Relationship between bystander and patient (family member)	16,194 (21.23)	16,279 (21.34)	0.60
Origin of OHCA, cardiac origin	43,846 (57.47)	43,987 (57.66)	0.47
***(CPR initiated by bystander)***			
Chest compression (yes)	30,070 (39.41)	30,222 (39.61)	0.43
Rescue breathing (yes)	11,242 (14.74)	11,275 (14.78)	0.82
Use of public-access AED (yes)	336 (0.44)	334 (0.44)	0.94
***(Life support by EMS personnel)***			
Emergency life-saving technician in ambulance (yes)	75,725 (99.26)	75,812 (99.37)	0.007
Medical doctor in ambulance (yes)	2,277 (2.98)	2,281 (2.99)	0.95
Advanced life support by MD (yes)	11,359 (14.89)	11,361 (14.89)	0.99
Time from call to arrival at scene (min) (SD)	7.39 (3.81)	7.42 (4.04)	0.15
Time from call to arrival at hospital (min) (SD)	35.01 (12.07)	35.27 (17.27)	0.001
First documented rhythm			
VF/pulseless VT	6,157 (8.07)	6,340 (8.31)	0.09
PEA/asystole	70,136 (91.93)	69,953 (91.69)	
Defibrillation by EMS personnel (yes)	8,848 (11.60)	9,043 (11.85)	0.12
Use of ALS devices (laryngeal mask/an adjunct airway/tracheal tubes)	55,024 (72.12)	54,946 (72.02)	0.66
Epinephrine use (yes)	1,297 (1.70)	955 (1.25)	<0.001
***(End points)***			
ROSC before hospital arrival (ROSC) (yes)	4,802 (6.29)	3,981 (5.22)	<0.001
1-mo survival after cardiac arrest (yes)	3,245 (4.25)	3,108 (4.07)	0.08
Cerebral performance category 1 mo after the event (good performance/moderate disability)	1,212 (1.59)	1,369 (1.79)	0.002
Overall performance category 1 mo after the event (no or mild neurological disability/moderate neurological disability)	1,207 (1.58)	1,364 (1.79)	0.002

ALS, advanced life support; PEA, pulseless electrical activity; SD, standard deviation; VT, ventricular tachycardia.

**Table 3:** Results of power calculations for all patients and propensity-matched patients.

Patient Type	Type I Error (α)	Power for Total Patient Group (1-β)	Power for Propensity-matched Patients (1-β)
ROSC	0.05	1.00	1.00
	0.01	1.00	1.00
1-mo survival	0.05	0.99	0.61
	0.01	0.93	0.37
CPC (1 or 2)	0.05	1.00	0.96
	0.01	1.00	0.87
OPC (1 or 2)	0.05	1.00	0.97
	0.01	1.00	0.91

## Results

### Patient Characteristics

During the 5 years of the study, 531,854 patients with OHCA met the inclusion criteria (Figure 1; Table 1). Of cases with missing values for study variables in Figure 1 (*n* = 4,272), 62 cases were missing the value for age. The age distributions of the remaining cases with values missing for study variables other than age (*n* = 4,210) and of all analyzed cases (*n* = 531,854) were not significantly different between the two groups. Statistically significant differences were observed between the LR and no-LR groups with respect to all variables in Table 1. While many differences likely were not clinically significant, one substantial difference was that patients who received LR solution were more likely to be intubated (74.23% versus 35.73%). One possible reason for this finding is that EMS staff who could insert an intravenous line tended to have greater clinical skills and experience, leading to more frequent prehospital endotracheal intubation. Patients with cardiac origin were more likely to receive LR solution than were patients with non-cardiac origin (21.58% versus 18.93%; χ^2^ = 266.56, *p<*0.001). Among patients with ROSC, time from the call to hospital arrival in the LR solution and the no-LR solution groups were 36.93±14.24 min and 40.43±25.91 min, respectively (*p*<0.0001).

### Prehospital IV LR Administration and Outcome in All Patients

**Figure 2:**
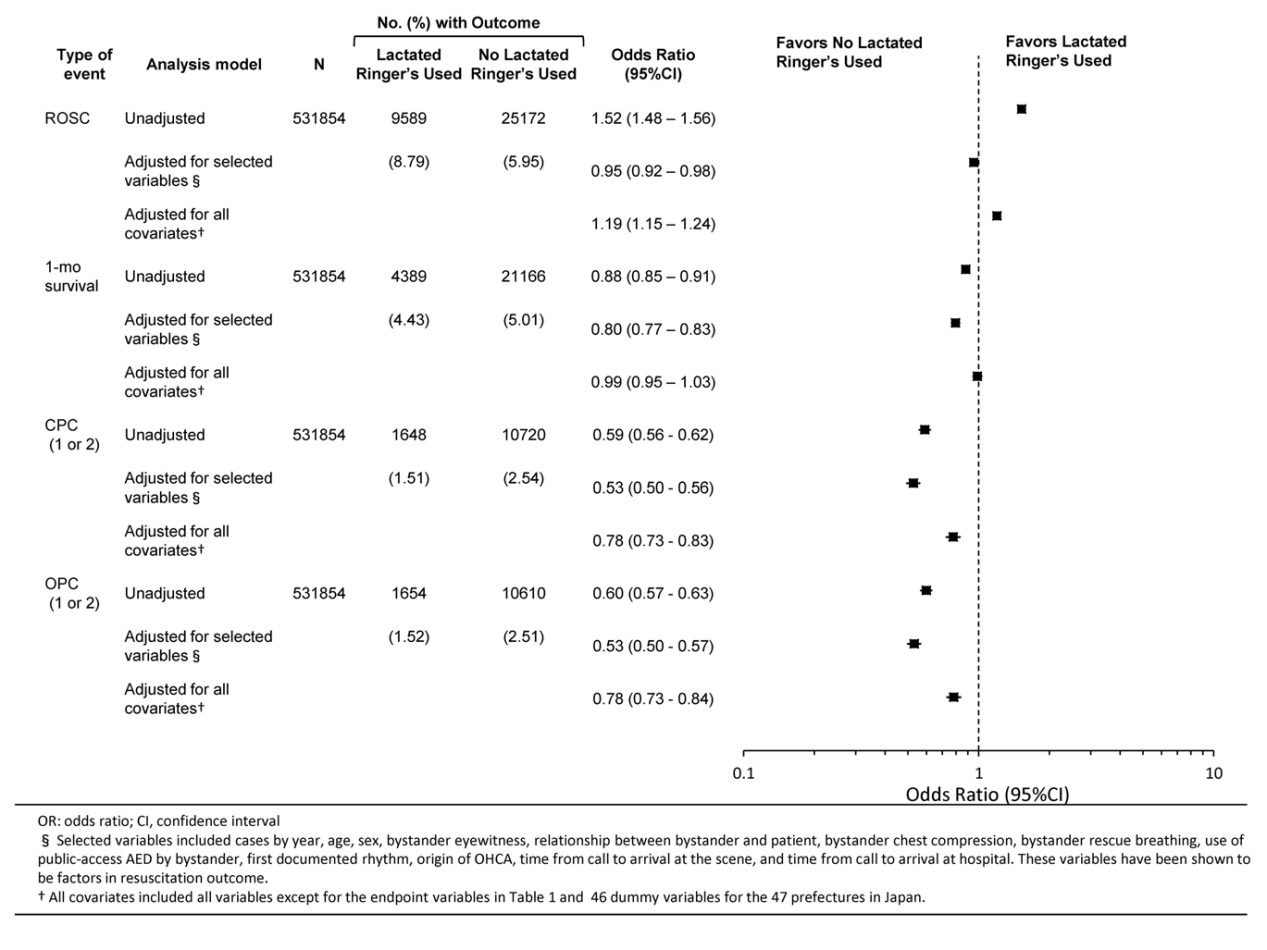
Results of unconditional logistic regression analyses comparing prehospital LR solution use versus no prehospital LR solution use in all patients with OHCA.

Figure 2 and Table 4 summarize patient outcomes on the basis of prehospital IV with LR and four types of outcome measures among all individuals. Administration of IV LR solution before hospital arrival was associated with a significantly higher rate of ROSC in the unadjusted model and fully adjusted model, but not in the model adjusted for selected variables (odds ratio [OR] = 1.521, 95% CI 1.484–1.559, *p*<0.001 in “unadjusted”; OR = 0.951, 95% CI 0.921–0.983, *p* = 0.003 in “adjusted for selected variables”; OR = 1.194, 95% CI 1.153–1.237, *p*<0.001 in “adjusted for all covariates”). IV LR administration was associated with a significantly lower rate of CPC at 1 month (OR = 0.880, 95% CI 0.852–0.909, *p*<0.001 in “unadjusted”; OR = 0.796, 95% CI 0.766–0.828, *p*<0.001 in “adjusted for selected variables”; OR = 0.986, 95% CI 0.946–1.029, *p* = 0.52 in “adjusted for all covariates”) and of OPC at 1 month (OR = 0.598, 95% CI 0.567–0.630, *p*<0.001 in “unadjusted”; OR = 0.531, 95% CI 0.499–0.565, *p*<0.001 in “adjusted for selected variables”; OR = 0.782, 95% CI 0.732–0.836, *p*<0.001 in “adjusted for all covariates”) in all three types of models. In the full-adjusted model, one-month overall survival was not significantly different between groups.

**Table 4:** Unconditional logistic regression analyses of prehospital LR solution use and outcomes among patients with OHCA: 2005–2009 national data in Japan (*n = *531,854).

Method	ROSC (Yes)		1-mo Survival (Yes)		CPC (1 or 2)		OPC (1 or 2)	
	OR (95% CI)	*p*-Value	OR (95% CI)	*p*-Value	OR (95% CI)	*p*-Value	OR (95% CI)	*p*-Value
Unadjusted	1.521 (1.484–1.559)	<0.001	0.880 (0.852–0.909)	<0.001	0.589 (0.559–0.621)	<0.001	0.598 (0.567–0.630)	<0.001
Adjusted for selected variables^a^	0.951 (0.921–0.983)	0.003	0.796 (0.766–0.828)	<0.001	0.527 (0.495–0.560)	<0.001	0.531 (0.499–0.565)	<0.001
Adjusted for all covariates^b^	1.194 (1.153–1.237)	<0.001	0.986 (0.946–1.029)	0.52	0.778 (0.728–0.832)	<0.001	0.782 (0.732–0.836)	<0.001

a Selected variables included cases by year, age, sex, bystander eyewitness, relationship between bystander and patient, bystander chest compression, bystander rescue breathing, use of public-access AED by bystander, first documented rhythm, origin of OHCA, time from call to arrival at the scene, and time from call to arrival at hospital. These variables have been shown to be factors in resuscitation outcome.

b All covariates included all variables except for the endpoint variables in Table 1 and 46 dummy variables for the 47 prefectures in Japan.

### Prehospital IV LR Administration and Outcome in Propensity-Matched Patients

The propensity scores ranged from 0.003 to 0.997, indicating that the probability of IV LR administration before hospital arrival would be between 0.003 and 0.997. This model yielded a *c* statistic of 0.85, indicating a strong ability to differentiate between IV LR before hospital arrival and other cases. In total, 76,293 patients receiving prehospital IV LR were matched to 76,293 patients not receiving prehospital IV LR (Table 2). No significant differences were detected between the LR use and no-LR use groups with respect to independent variables except for “cases by year” (*p* = 0.002), “bystander eyewitness” (*p* = 0.03), “emergency life-saving technician in ambulance” (*p* = 0.007), “time from call to hospital arrival” (*p* = 0.001), and “epinephrine use” (*p*<0.001).

The number of 1-month survivors in the propensity-matched LR solution and no-LR solution groups were 1,876 (43.30%) and 1,721 (46.83%), respectively (*p = *0.001). The numbers of patients in CPC categories 1 or 2 in the LR solution and no-LR solution groups were 937 (21.57%) and 1,042 (28.35%), respectively (*p*<0.0001). The numbers of patients in OPC categories 1 or 2 in the LR solution and no-LR solution groups were 933 (21.48%) and 1,040 (28.30%), respectively (*p*<0.0001).

**Figure 3:**
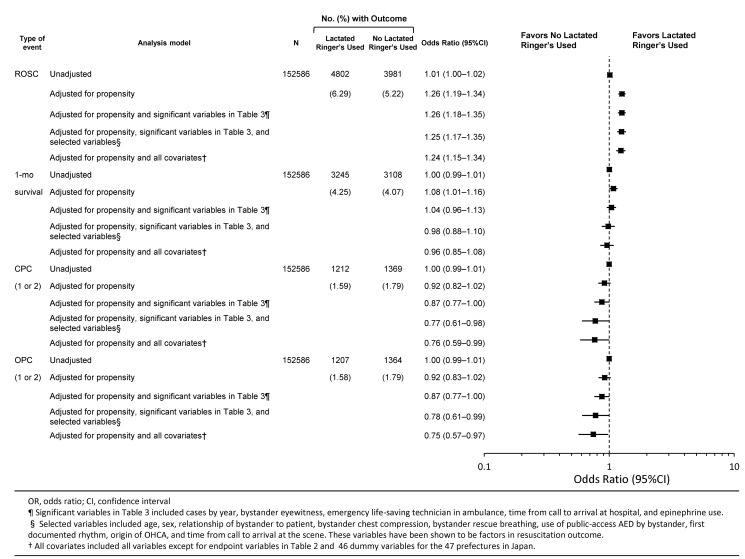
Results of conditional logistic regression analyses comparing prehospital LR solution use versus no prehospital LR solution use in propensity-matched patients with OHCA.

Figure 3 and Table 5 summarize survival outcomes on the basis of prehospital IV administration of LR solution among propensity-matched patients. Prehospital IV LR administration was associated with increased likelihood of ROSC in all five propensity analysis models (OR = 1.011, 95% CI 1.001–1.021, *p = *0.04 in “unadjusted”; OR = 1.264, 95% CI 1.193–1.339, *p*<0.001 in “adjusted for propensity”; OR = 1.262, 95% CI 1.182–1.345, *p*<0.001 in “adjusted for propensity and significant variables in Table 2”; OR = 1.254, 95% CI 1.166–1.349, *p*<0.001 in “adjusted for propensity, significant variables in Table 2, and selected variables”; OR = 1.239, 95% CI 1.146–1.339, *p*<0.001 in “adjusted for propensity and all covariates”). However, prehospital IV LR administration was associated with a reduced likelihood of CPC category 1 or 2 at 1 month and OPC category 1 or 2 at 1 month in propensity models (OR = 0.873, 95% CI 0.7660–0.995, *p* = 0.04; and OR = 0.873, 95% CI 0.766–0.995, *p = *0.04, respectively), for propensity, significant variables in Table 2, and selected variables (OR = 0.773, 95% CI 0.609–0.982, *p* = 0.04; and OR = 0.777, 95% CI 0.611–0.988, *p = *0.04, respectively), and for propensity and all covariates (OR = 0.764, 95% CI 0.589–0.992, *p* = 0.04; and OR = 0.746, 95% CI 0.573–0.971, *p = *0.03, respectively). In the fully adjusted models, one-month overall survival was not significantly different between the groups.

**Table 5:** Conditional logistic regression analysis of prehospital LR solution use and outcomes among propensity-matched patients with OHCA: 2005–2009 national data in Japan (*n = *152,586).

Method	ROSC (yes)		1-mo Survival (Yes)		CPC (1 or 2)		OPC (1 or 2)	
	OR (95% CI)	*p*-Value	OR (95% CI)	*p*-Value	OR (95% CI)	*p*-Value	OR (95% CI)	*p*-Value
Unadjusted	1.011 (1.001–1.021)	0.04	1.002 (0.992–1.012)	0.73	0.998 (0.988–1.008)	0.69	0.998 (0.988–1.008)	0.69
Adjusted for propensity	1.264 (1.193–1.339)	<0.001	1.080 (1.009–1.156)	0.03	0.915 (0.823–1.017)	0.10	0.919 (0.827–1.022)	0.12
Adjusted for propensity and significant variables in Table 2 a	1.262 (1.182–1.345)	<0.001	1.041 (0.963–1.125)	0.31	0.873 (0.766–0.995)	0.04	0.873 (0.766–0.995)	0.04
Adjusted for propensity, significant variables in Table 2, and selected variables^b^	1.254 (1.166–1.349)	<0.001	0.981 (0.877–1.098)	0.74	0.773 (0.609–0.982)	0.04	0.777 (0.611–0.988)	0.04
Adjusted for propensity and all covariates^c^	1.239 (1.146–1.339)	<0.001	0.960 (0.854–1.078)	0.49	0.764 (0.589–0.992)	0.04	0.746 (0.573–0.971)	0.03

a Significant variables in Table 2 included cases by year, bystander eyewitness, emergency life-saving technician in ambulance, time from call to arrival at hospital, and epinephrine use.

b Selected variables included age, sex, relationship of bystander to patient, bystander chest compression, bystander rescue breathing, use of public-access AED by bystander, first documented rhythm, origin of OHCA, and time from call to arrival at the scene. These variables have been shown to be factors in resuscitation outcome.

c All covariates included all variables except for endpoint variables in Table 2 and 46 dummy variables for the 47 prefectures in Japan.

We compared time from the call to hospital arrival in the LR solution with that in the no-LR solution groups. The times were 36.93±14.24 min and 40.43±25.91 min, respectively (*p*<0.0001). Although on average the LR solution group was transported more quickly to the emergency department, and, thus, definitive care could be performed more rapidly, survival at 1 month was not different between the groups and clinical outcomes in the IV LR group were worse.

## Discussion

We found that prehospital IV administration of LR solution to patients with OHCA was independently associated with decreased 1-month survival with minimal neurological impairment and disability (i.e., CPC category 1 or 2, OPC category 1 or 2), as well as with increased ROSC before hospital arrival (Tables 2 and 5). Our findings are derived from national registry data, and the sample size for the propensity analysis was ample except for the analysis of 1-mo survival (Table 4). On the basis of the propensity analysis controlling for selection bias and confounding factors, prehospital intravenous loading with LR solution does not appear to be beneficial for patients with OHCA.

We believe that the present findings could have significant theoretical and practical implications. There are two possible explanations for the association between prehospital intravenous loading with LR solution and decreased likelihood of survival with minimal neurological or physical impairment. First, 18 types of LR solution products were used during the study period, and the pH of the solutions was 6.7. Although LR solutions with an acidity regulator were used in some cases, the proportion of such products to total LR solution consumption in Japan from 2006 to 2009 was 0.9% [23]. Based on previous studies' mean time from call to hospital arrival [24],[28],[29] and mean lactate concentration at hospital admission (i.e., 7.49–12.57 mmol/l) (Table S1), as well as mean time from call to hospital arrival in the present study (35.18 min) (Table 2), most patients with OHCA would have developed lactic acidosis by the time of hospital admission.. Thus, prehospital LR use could worsen advancing acidemia in patients with OHCA, and adverse consequences of acidemia can occur independently of whether the acidemia is of metabolic, respiratory, or mixed origin [30]. Of the major acid-base disorders, the effects on the cardiovascular system are particularly pernicious and include decreased cardiac output and decreased arterial blood pressure [30]. Furthermore, higher lactate concentration at hospital admission was related to 1-mo survival with serious neurological impairment (CPC category ≥3) [28],[31].

Since hyponatremia may play a role as well, we also considered this possibility. Although the concentration of Na in the LR solutions was 130 mEq/l, the total amount of LR loading was estimated to be 200–300 ml at most. Thus, the hyponatremic effect of LR in the blood would be extremely limited.

A third explanation may be considered. Lactic acid is released during cellular hypoxia. Lactate is a marker for tissue hypoxia, and thus prognosis, but by itself is not toxic and is used as a cellular fuel. The LR used in this study has a tonicity of 275 mOsm/l. When measured by freezing point depression, the osmolarity is 254 mOsm/l because of incomplete ionization of solutes in LR [32]. Despite its slight hypotonicity, when used clinically, infusion of large volumes of LR produces only a small transient change in serum osmolality. Since the osmolarity of these solutions is slightly lower than 280 mOsm/l, the hypotonicity of the LR solution used may be responsible [32]. In addition, although the pH of these solutions is about 6.5 ex vivo, these solutions are alkalinizing, since lactate is oxidized to bicarbonate in the liver. Therefore, the alkalinizing effect of the RL solution may be deleterious in the present study [33],[34]. However, OHCA patients have already developed lactic acidosis at the time of hospital admission in many studies, and this explanation might not be consistent with previous findings (Table S1). In conclusion, given that lowered blood pH is harmful to homeostasis [30], we believe that the worse outcomes in the LR group may be a result of lower pH fluid loading, but we acknowledge that other explanations are possible.

No studies have reported the efficacy of solutions used to resuscitate patients with OHCA. Specifically, Bender et al. reported that hypertonic saline during CPR for adult patients with OHCA was not related to ROSC [35]. Normal saline at 4°C or LR solution at 4°C during CPR in adult patients with OHCA was not related to ROSC before hospital arrival [36],[37]. Our study is the first to show, to our knowledge, that prehospital IV loading with LR solution was independently associated with increased ROSC before hospital arrival. Five analytical models were used in propensity-matched patients, and the propensity findings were consistent (Table 5). We believe that intravenous LR solution infusion leads to higher hydrostatic pressure and higher blood pressure, which could result in increased ROSC before hospital arrival.

There are several notable points in our study. First, we conducted an analysis of the association between prehospital use of LR solution and outcomes in the entire sample (Table 4) as well as the propensity analysis. Prehospital IV LR use was associated with a reduced likelihood of CPC and OPC at 1 month, which was consistent with the results obtained for propensity-matched patients (Table 5). However, there were discrepancies between findings based on all individuals and propensity-matched patients with respect to associations between prehospital LR solution use and ROSC and with 1-month survival. Generally, an observational study cannot be free from selection bias and confounding factors [16]. These discrepancies may have been due to selection bias and confounding factors in the analyses based on all individuals. Second, in a previous study we analyzed the Utstein registry data between 2005 and 2008 in Japan and reported that the use of prehospital epinephrine was significantly associated with an increased chance of ROSC before hospital arrival, but a decreased chance of having a good functional outcome 1 mo after the event [18]. Although there is a difference between the data analyzed in the previous and the present studies (i.e., Utstein data for 2005–2008 versus 2005–2009), the association of epinephrine with the outcome variables seems to be greater than that of LR solution. Specifically, the ORs of prehospital epinephrine use were between 1.91 and 2.51 for ROSC before hospital arrival [18], whereas the ORs of prehospital LR solution use were between 1.01 and 1.26 for ROSC before hospital arrival (Table 5). The ORs of prehospital epinephrine use were between 0.21 and 0.41 and between 0.23 and 0.43 for CPC (1, 2) and OPC (1, 2), respectively [18], whereas those of prehospital LR solution use were between 0.76 and 0.87 and between 0.75 and 0.87, respectively (Table 5). In the current study, after propensity matching, a difference between the LR and No-LR groups remained with respect to epinephrine use, with the LR group receiving epinephrine more frequently than the no-LR group (1.70% versus 1.25%, *p* = 0.00) (Table 2). This discrepancy might be why the propensity models that did not control for prehospital epinephrine use found no association between prehospital LR solution use and CPC (1, 2) or OPC (1, 2) (Table 5). Third, because more patients survived with LR, more might die later and/or have worse non-mortality outcomes. We verified this point by comparing the association of prehospital LR solution use with resuscitation outcome among the LR solution and no-LR solution groups based on the ratio of relative risks (RRR) (Table S2) [38]. A third study using this database was recently published that compared bag-valve-mask ventilation with advanced airway management and found that patients receiving bag-valve-mask ventilation had better neurologically favorable survival [39]. This factor was accounted for in our variable Use of ALS devices (laryngeal mask/an adjunct airway/tracheal tubes).

Several limitations and caveats to our study must be acknowledged. First, data on in-hospital CPR after hospital arrival were not included in analyses. It is possible that our findings may have been due to a difference in in-hospital resuscitation, such as hypothermia [40] and mechanical chest compression devices [41], among the LR solution and no-LR solution groups. Although the quality of in-hospital resuscitation might influence 1-month survival, we could not control for the effects of such factors. Second, prehospital LR solution use was not assigned randomly. We performed a propensity analysis and made a rigorous adjustment for selection bias and confounding factors [11]; nevertheless, we acknowledge that observational studies can only partially control and adjust for factors actually measured, whereas randomized allocation can control both known and unknown confounding factors and avoid introducing bias. Third, fluid administration with LR is not the standard of care, or is not part of the OHCA protocol, in many other countries. Therefore, the generalizability of these results might be limited to countries where fluid administration with LR is the standard of care for OHCA patients. Future studies will need to determine whether administration of fluids other than LR is associated with beneficial outcomes.

In summary, we found that prehospital IV loading with LR solution was independently associated with a decreased likelihood of 1-month survival with minimal neurological or physical impairment. Prehospital IV loading with LR solution was associated with an increased likelihood of ROSC before hospital arrival; while 1 month survival varied depending on the analysis. Our findings should be verified by studies that include data on in-hospital resuscitation.

## Supporting Information

Table S1 Systematic literature review on arterial lactate concentrations at hospital admission among patients with OHCA.
(DOC)

Table S2 Comparison of two estimated relative risks of prehospital use of LR solution on resuscitation outcome.
(DOC)

Text S1 STROBE checklist.
(DOC)

## Abbreviations

AED: automated external defibrillator
CPC: Cerebral Performance Category
EMS: emergency medical service
LR: lactated Ringer's
OHCA: out-of-hospital cardiac arrest
OPC: Overall Performance Category
OR: odds ratio
ROSC: return of spontaneous circulation
VF: ventricular fibrillation
