# Supplementary material for: Prehospital Lactated Ringer's Solution Treatment and Survival in Out-of-Hospital Cardiac Arrest: A Prospective Cohort Analysis
Source: PLoS Med. 2013 Feb 19;10(2):e1001394. doi: 10.1371/journal.pmed.1001394 (PMC3576391; doi:10.1371/journal.pmed.1001394)
Supplement: Table S1 — Systematic literature review on arterial lactate concentrations at hospital admission among patients with OHCA. (DOC) [file pmed.1001394.s001.doc]

Online supporting information

**Table S1. Systematic literature review on arterial lactate concentrations at hospital admission among patients with OHCA.**

| No. | Time of study /authors | Place | Time from call to scene/hospital arrival (min) | Characteristics of patients with OHCA | Lactate concentration  (mmol/*l* or mg/dl) | pH of arterial blood |
| --- | --- | --- | --- | --- | --- | --- |
| 1 | -- /Sugita^1^ | Saitama prefecture, Japan (single-center study) |  | Patients admitted to the emergency unit (*n =* 61) | ROSC group (*n =* 18) : 16.20 ± 5.28  non-ROSC group (*n =* 43): 13.50 ± 5.21 | ROSC group (*n =* 18): 7.08 ± 0.19  non-ROSC group (*n =* 43)  7.04 ± 0.27 |
| 2 | December 2004–April 2005/Kuroki et al.^2^ | Tokyo, Japan (single-center study) | Hospital arrival:  32 ± 5 (ROSC group),  34 ± 9 (non-ROSC group) | Consecutive patients admitted to the emergency unit whose initial rhythm was other than PEA/asystole. Arterial blood was drawn within 5 minutes after hospital admission. (*n =* 43) | ROSC group (*n =* 5):  97 ± 5 (mg/dl)  1 non-ROSC group (*n =* 38): 127 ± 48 (mg/dl) | ROSC group (*n =* 5): 7.06 ± 0.10  non-ROSC group (*n =*38): 6.95 ± 0.20 |
| 3 | April 2000–March 2009/Yamamoto et al.^3^ | Tokyo, Japan (single-center study) |  | Patients who met the institutional criteria for TH out of 1,996 consecutive OHCA patients transferred to the emergency unit. (*n =* 39)  Median ages were 61 (CPC, 1/2) and 61 yr (CPC, ≥3) | CPC = 1/2 (*n =* 18):  6.2 (IQR, 2.6–8.1)  CPC ≥3 (*n =* 21)  9.6 (IQR, 7.7–11.8) |  |
| 4 | August 2000–October 2000 /Masui et al.^4^ | Kanagawa prefecture, Japan (single-center study) |  | Consecutive patients admitted to the emergency unit whose arterial blood was drawn at hospital admission (*n =* 80)  Mean age of ROSC patient group (*n =* 14) was 76 years, and that of dead arrival group (*n =* 66) was 74 years. | ROSC group (*n =* 14): 7.63 ± 2.25  non-ROSC group (*n =* 66):  10.26 ± 4.8 |  |
| 5 | February 2007–July 2010 /Cho et al.^5^ | Inchon City, South Korea (single-center study) |  | Patients (≥18 years) who were successfully resuscitated after nontraumatic OHCA and then treated with TH (*n =* 117) | CPC = 1/2 (*n =* 34):  8.75 (IQR, 6.60–11.30)  CPC≥3 (*n =* 83)  9.40 (7.40–11.80) (IQR) |  |
| 6 | April 2009–June 2010/Ito et al.^6^ | Osaka, Japan (single-center study) | Hospital arrival:  40 (interquartile range [IQR], 31–49) | Consecutive patients (≥18 years) admitted to the emergency unit. (*n =* 92) | CPC = 1/2 (*n =* 13):  8.9 ± 4.7  CPC ≥3 (*n =* 79):  12.5 ± 4.6 |  |
| 7 | May 2007–March 2009 /Shinozaki et al.^7^ | Chiba, Japan (multiple-center study) |  | Consecutive patients (≥18 years) admitted to the three emergency units (715 ± 15 yr) who gained ROSC. (*n =* 98) | CPC = 1/2 (*n =* 10):  9.2 (IQR, 2.6–11.5)  CPC ≥3 (*n =* 88):  12.1 (IQR, 9.5–14.0) |  |
| 8 | 1999–2003/Adrie et al.^8^ | Saint Denis, France (multiple-center study) | No-flow (duration before CPR) interval: 6 (IQR, 3–10) | Consecutive patients (≥18 years) admitted to the emergency unit [age, 55 yr, (IQR, 47–69)] who recovered blood pressure and pulse for more than 1 hr (*n =* 130) | 6.7 (IQR, 3.7–11) |  |
| 9 | 2003–2005/Adrie et al.^8^ | Saint Denis, Troyes, Paris, France (multiple-center study) | No-flow (duration before CPR) interval: 7 (IQR, 3–10) | Consecutive patients (≥18 years) admitted to the four emergency units [age, 56 yr (IQR, 45–69)] who recovered blood pressure and pulse for more than 1 hr (*n =* 130) | 6.2 (IQR, 3.2–11.3) |  |
| 10 | --/Pynnönen et al.^9^ | Tampere, Finland (single-center study) |  | Consecutive OHCA patients (≥18 years) with witnessed CA, shockable rhythm, cardiac origin arrest, ROSC within 30 min from the arrest (*n =* 8) | 1.7 (IQR, 1.4–2) |  |
| 11 | November 2004–December 2006/Okasanen et al.^10^ | Helsinki, Finland (multiple-center study) | Time to first responding unit:  8 (IQR, 6–9) | Consecutive patients (≥18 years) with shockable rhythm, cardiac origin, witnessed arrest, BLS within 15 minutes from the arrest, ROSC less than 35 minutes from the arrest, nonresponsive at admission. (*n =* 90) | 4.5 (IQR, 2.4–6.5) |  |
| 12 | February 2002–July 2003/Pene et al.^11^ | Paris, France (single-center study) | No-flow (duration before CPR) interval: 3 (IQR, 3–8)  Low-flow interval (duration of CPR): 10 (IQR, 5–19)  ROSC duration:  15 (IQR, 5–25) | Consecutive patients with OHCA who regained ROSC after resuscitation in the field. (*n =* 64) | 4 (IQR, 2.5–6.4) |  |
| 13 | September 2004–September 2005/Kim et al.^12^ | Inchon, Korea (single-center study) | Mean time from arrest to CPR:  8.00 ± 7.90 (range, 1–30) | Consecutive patients (≥16 years) admitted to the IUC. (*n =* 30) | 7.49 ±2.59 (1.9–14.1) |  |
| 14 | January 2003–December 2008/Dumas et al.^13^ | Paris, France (multiple-center study) | Time from collapse to BLS:  <5 (*n =* 223, 53%) ≥5 (*n =* 210, 51%) | Consecutive patients who recovered ROSC before hospital arrival. (*n =* 435) | 4.9 (IQR, 2–7) |  |
| 15 | February 1999–April 2000, December 2011–July 2002/Adrie et al.^14^ | Paris, France (multiple-center study) | Time from collapse to BLS:  6 (IQR, 3–10) | Consecutive patients who recovered blood pressure and pulse for more than 1 hr. (*n =* 67) | 5.2 (IQR, 2.6–10) |  |
| 16 | December 2004–October 2006/Oddo et al.^15^ | Lausanne, Switzerland (single-center study) |  | Consecutive patients (<80 years) who were admitted to ICU for persistent coma after OHCA (*n =* 74). Median age was 61 yr (IQR, 51–68). | 10.0 (IQR, 7.4–13.1) |  |
| 17 | January 2000–December 2009/Dumas et al.^16^ | Paris, France (multiple-center study) | No-flow (duration before CPR) interval: 4 (IQR, 0–10) | Consecutive patients with OHCA who regained ROSC after resuscitation on the field. (*n =* 1145) | 4.5 (IQR, 2.2–8.5) |  |
| 18 | February 1999–November 2006/Adrie et al.^17^ | Saint Denis, Massy, Le Kremlin Bicetre, France  (multiple-center study) | No-flow (duration before CPR) interval: CPC = 1, 2: 5 (IQR, 1–10)  CPC = 3, 4:  10 (IQR, 5–12)  CPC = 5: 9 (IQR, 5–11) | Consecutive patients with OHCA who regained ROSC after resuscitation in the field. (*n =* 246) | CPC = 1, 2 (*n =* 36):  4 (IQR, 1.7–8.9)  CPC = 3, 4 (*n =* 170):  10 (IQR, 5.8–14.7)  CPC = 5 (*n =* 40):  11 (IQR, 6.8–15.9) |  |
| 19 | February–December  2005/Tsai et al.^18^ | Taipei, Taiwan (single-center study) |  | Consecutive non-traumatic patients (>18 yr) with OHCA admitted to the university hospital (*n =* 56). Mean age was 73.4 ± 14.7 years. | AIVR group (*n =* 8): 7.63 (IQR, 5.92–9.34)  Non-AIVR group (*n =* 48):  8.08 (IQR, 6.29–9.83) | AIVR group: 7.11 (6.94–7.23)  Non-AIVR group: 7.08 (6.96–7.22) |
| 20 | September 2006–August 2009/Cocchi et al.^19^ | Boston, USA (single-center study) | No-flow (duration before CPR) interval:  21.68 ± 17 | Consecutive OHCA patients (>18 yr) who gained ROSC in the field, were admitted to the hospital, and had a non-contract CT scan within the first 24 hr. Mean age was 63.8 ± 18.7 yr (*n =* 51). | 5.6 ± 4.2 |  |
| 21 | January 2007–December 2008/Lee et al.^20^ | Inchon, Korea (single-center study) |  | Elderly patients with OHCA (≥60 years) (*n =* 38) and younger OHCA patients (<60 years). (*n =* 88) | Elderly patients (≥60 yr) (*n =* 38): 7.75 ± 2.87  Younger patients (<60 yr) (*n =* 50): 10.7 ± 3.58. | Elderly pts (≥60 yr):  7.16 ± 0.18  Younger pts (<60 yr):  7.00 ± 0.13 |
| 22 | March 2002–June 2003/Hékimian et al.^21^ | Saint Denis and Massy, France (multiple-center study) | Time from collapse to first-response life support:  Survivors (*n =* 4):  4.5 (IQR, 2–7)  Dead from early refractory shock  (*n =* 10):  12 (IQR, 10-15)  Dead from neurologic dysfunction (*n =* 9):  *8* (IQR, 5–11) | Consecutive OHCA patients (>16 yr) who gained ROSC in the field, were admitted to the hospital, and showed stable hemodynamic condition at ICU admission with or without vasopressor agents (*n =* 33). Median ages were 58 yr (range, 49–65) for “Survivors,” 51 yr (range, 45–54) for “dead from early refractory shock,” and 48 yr (range, 38–57) for “dead from neurologic dysfunction.” | Survivors(*n =* 4):  7.4 (*3*–) 11  Dead from early refractory shock (*n =* 10):  14 (8.5–16)  Dead from neurologic dysfunction (*n =* 19):  7.7 (4–11) |  |
| 23 | 2005–2007/Pfeifer et al.^22^ | Jena, Germany (single-center study) | Time of anoxia:  Survivors (*n =* 9):  3 ± 1.9  Non-survivors (*n =* 9): 7.2 ± 7.9 | Consecutive OHCA patients who gained ROSC were admitted to ICU and were treated with TH. The patients had continuous sinus rhythm and stabilized cardiovascular circulation, and survived at least 48 h (*n =* 18). Mean age was 62.3 ± 13.2 years. | Survivors (*n =* 9):  3.8 ± 3.8  Non-survivors *(n =* 9):  7.7 ± 2.5 |  |
| 24 | --/Busch et al.^23^ | Norway (case report) | From call to scene arrival: 9 | A 26-year old female without an eyewitness, no bystander CPR, asystole initial rhythm, and no cardiac origin (*n =* 1) | 17.1 | 6.9 |

**Note.** A MEDLINE search for the English literature and ICHUSHI (“Igaku Chuo Zasshi”) search for Japanese literature were performed to obtain findings concerning arterial lactate concentrations at hospital admission among patients with out-of-hospital cardiac arrest. “OHCA” + “lactate” were used as keywords to retrieve potentially related articles. After reviewing the retrieved articles, relevant articles are summarized in the table.

**Literature Cited**

1. Sugita M. Can lactic acidosis predict the prognosis of patients delivered as out-of-hospital cardiac arrest? ICU&CCU 2003; 27(10):917-921. (in Japanese)

2. Kuroki Y, Ikeda T, Ikeda K, Mukojima K, Yokoyama T, Yoshikawa K. One-way association between arterial lactate concentration and ROSC among patients with OHCA. Journal of Japanese Society for Emergency Medicine 2007; 10:393-396. (in Japanese)

3. Yutaka Y, Tomio J, Tanabe T, Sugiyama K, Kuroki N, Abe H, Akashi A, Hamabe Y. Factors associated with neurological outcome in the resuscitated survivors of out-of-hospital cardiac arrest after mild therapeutic hypothermia. ICU&CCU 2010; 34(9):741-746. (in Japanese)

4. Masui Y, Ishiyama A, Ida K, Kobayashi H, Kobayashi M, Sakaino T, Nishio S, Ikehara Y, Noda S, Akashi K. Prediction of successful resuscitation using lactate concentration at hospital admission among patients with OHCA. Journal of Japanese Society for Emergency Medicine 2001; 4:196. (in Japanese)

5. Cho YM, Lim YS, Yang HJ, Park WB, Cho JS, Kim JJ, Hyun SY, Lee MJ, Kang YJ, Lee G. Blood ammonia is a predictive biomarker of neurologic outcome in cardiac arrest patients treated with therapeutic hypothermia. The American Journal of Emergency Medicine (in press). (Available online 26 December 2011)

6. Ito N, Nanto S, Nagao K, Hatanaka T, Nishiyama K, Kai T. Regional cerebral oxygen saturation on hospital arrival is a potential novel predictor of neurological outcomes at hospital discharge in patients with out-of-hospital cardiac arrest. Resuscitation 2011; 83:46-50.

7. Shinozaki K, Oda S, Sadahiro T, Nakamura M, Hirayama Y, Watanabe E, Tateishi Y, Nakanishi K, Kitamura N, Sato Y, Hirasawa H. Blood ammonia and lactate levels on hospital arrival as a predictive biomarker in patients with out-of-hospital cardiac arrest. Resuscitation 2011; 82(4):404–409.

8. Adrie C, Cariou A, Mourvillier B, Laurent I, Dabbane H, Hantala F, Rhaoui A, Thuong M, Monchi M. Predicting survival with good neurological recovery at hospital admission after successful resuscitation of out-of-hospital cardiac arrest: the OHCA score. European Heart Journal 2006; 27:2840-2845.

9. Pynnönen L, Falkenbach P, Kämäräinen A, Lönnrot K, Yli-Hankala A, Tenhunen J. Therapeutic hypothermia after cardiac arrest-cerebral perfusion and metabolism during upper and lower threshold normocapnia. Resuscitation 2011; 82:1174–1179.

10. Oksanen T, Tiainen M, Skrifvars MB, Varpula T, Kuitunen A, Castrén M, Pettilä V. Predictive power of serum NSE and OHCA score regarding 6-month neurologic outcome after out-of-hospital ventricular fibrillation and therapeutic hypothermia. Resuscitation 2009; 80:165–170.

11. Pene F, Hyvernat H, Mallet V, Cariou A, Carli P, Spaulding C, Dugue MA, Mira JP. Prognostic value of relative adrenal insufficiency after out-of-hospital cardiac arrest. Intensive Care Medicine 2005; 31:627–633.

12. Kim JJ, Lim YS, Shin JH, Yang HJ, Kim JK, Hyun SY, Rhoo I, Hwang SY, Lee G. Relative adrenal insufficiency after cardiac arrest: impact on postresuscitation disease outcome. The American Journal of Emergency Medicine 2006; 24:684–688.

13. Dumas F, Cariou A, Manzo-Silberman S, Grimaldi D, Vivien B, Rosencher J, Empana JP, Carli P, Mira JP, Jouven X, Spaulding C. Immediate percutaneous coronary intervention is associated with better survival after out-of-hospital cardiac arrest: insights from the PROCAT (Parisian Region Out of Hospital Cardiac Arrest) registry. Circulation. Cardiovascular Interventions: 2010; 3:200–207.

14. Adrie C, Monchi M, Laurent I, Um S, Yan SB, Thuong M, Cariou A, Charpentier J, Dhainaut JF. Coagulopathy after successful cardiopulmonary resuscitation following cardiac arrest: implication of the protein C anticoagulant pathway. Journal of the American College of Cardiology 2005; 46:21-28.

15. Oddo M, Ribordy V, Feihl F, Rossetti AO, Schaller MD, Chioléro R, Liaudet L. Early predictors of outcome in comatose survivors of ventricular fibrillation and non-ventricular fibrillation cardiac arrest treated with hypothermia: A prospective study. Critical Care Medicine 2008; 36(8):2296-2301.

16. Dumas F, Grimaldi D, Zuber B, Fichet J, Charpentier J, Pène F, Vivien B, Varenne O, Carli P, Jouven X, Empana JP, Cariou A. Is hypothermia after cardiac arrest effective in both shockable and nonshockable patients? : insights from a large registry. Circulation 2011; 123:877-886.

17. Adrie C, Haouache H, Saleh M, Memain N Laurent I, Thuong M, Darques L, Guerrini P, Monchi M. An underrecognized source of organ donors: patients with brain death after successfully resuscitated cardiac arrest. Intensive Care Medicine 2008; 34:132-137.

18. Tsai M, Huang C, Chen H, Hsieh C, Chang W, Hsu C, Ma M H, Chen S, Chen W. Postresuscitation accelerated idioventricular rhythm: a potential prognostic factor for out-of-hospital cardiac arrest survivors. Intensive Care Medicine 2007; 33:1628-1632

19. Cocchi M, Lucas J, Salciccioli J, Carney E, Herman S, Zimetbaum P, Donnino M. The role of cranial computed tomography in the immediate post-cardiac arrest period. Internal and Emergency Medicine 2010; 5:553-538.

20. Lee S, Lim Y, Cho J, Kim J, Kim J, Kim J, Hyun S, Yang H, Lee G. Outcome and factors associated with mortality of elderly who treated with therapeutic hypothermia after out-of-hospital cardiac arrest: comparative study between more and less than 60 years old. Journal of the Korean Geriatrics Society. 2009; 13(4):203-214.

21. Hékimian G, Baugnon T, Thuong M, Monchi M, Dabbane H, Jaby D, Rhaoui A, Laurent I, Moret G, Fraisse F, Adrie C. Cortisol levels and adrenal reserve after successful cardiac arrest resuscitation. Shock 2004; 22(2):116-119.

22. Pfeifer R, Hopfe J, Ehrhardt C, Goernig M, Figulla HR, Voss A. Autonomic regulation during mild therapeutic hypothermia in cardiopulmonary resuscitated patients . Clinical Research in Cardiology 2011; 100:797-805.

23. Busch M, Søreide E. Successful use of therapeutic hypothermia in an opiate induced out-of-hospital cardiac arrest complicated by severe hypoglycaemia and amphetamine intoxication: a case report. Scandinavian Journal of Trauma, Resuscitation and Emergency Medicine 2010; 18(4):1–3.
