# Supplementary material for: Prehospital Lactated Ringer's Solution Treatment and Survival in Out-of-Hospital Cardiac Arrest: A Prospective Cohort Analysis
Source: PLoS Med. 2013 Feb 19;10(2):e1001394. doi: 10.1371/journal.pmed.1001394 (PMC3576391; doi:10.1371/journal.pmed.1001394)
Supplement: Table S2 — Comparison of two estimated relative risks of prehospital use of LR solution on resuscitation outcome. (DOC) [file pmed.1001394.s002.doc]

Online supporting information

**Table S2. Comparison of two estimated relative risks of prehospital use of LR solution on resuscitation outcome.**

|  | ROSC (+) | | RR | (95% CI) | ROSC (-) | | RR | (95% CI) | RRR | (95% CI) |
| --- | --- | --- | --- | --- | --- | --- | --- | --- | --- | --- |
| LR (+) | n | (%) |  |  | n | (%) |  |  |  |  |
| 1-month survival | 2921 | (30.42) | 0.60 | (0.58–0.62) | 1926 | (1.93) | 0.90 | (0.86–0.95) | 0.67 | (0.63–0.71) |
| CPC (1 or 2) | 1270 | (13.23) | 0.39 | (0.37–0.41) | 383 | (0.38) | 0.69 | (0.62–0.77) | 0.57 | (0.50–0.64) |
| OPC (1 or 2) | 1270 | (13.23) | 0.39 | (0.37–0.42) | 389 | (0.39) | 0.71 | (0.64–0.79) | 0.56 | (0.49–0.63) |
| RR: relative risk; CI: confidence interval; RRR: ratio of relative risk  Note. Relative risks of LR solution on resuscitation outcome in ROSC(+) and ROSC(-) groups were compared by calculating the ratio of relative risk (RRR).  Reference. Altman DG, Bland JM. Interaction revisited: the difference between two estimates. BMJ 2003; 326(25):219. | | | | | | | | | | |
