# Supplementary material for: Prehospital Lactated Ringer's Solution Treatment and Survival in Out-of-Hospital Cardiac Arrest: A Prospective Cohort Analysis
Source: PLoS Med. 2013 Feb 19;10(2):e1001394. doi: 10.1371/journal.pmed.1001394 (PMC3576391; doi:10.1371/journal.pmed.1001394)
Supplement: Text S1 — STROBE checklist. (DOC) [file pmed.1001394.s003.doc]

*STROBE Statement – Checklist of items that should be included in reports of* ***cohort studies***

Corresponding author: Akihito Hagihara

Title: Prehospital lactated Ringer's solution loading and survival in out-of-hospital cardiac arrest

Manuscript ID: PMEDICINE-D-12-02631R1

|  | **Item**  **No** | ***Recommendation* & Responses** |
| --- | --- | --- |
| **Title and abstract** | 1 | *(a) Indicate the study’s design with a commonly used term in the title or the abstract*  [Title] Prehospital lactated Ringer’s solution treatment and survival in out-of-hospital cardiac arrest: A prospective cohort analysis. |
|  |  | *(b) Provide in the abstract an informative and balanced summary of what was done and what was found*  Background  No studies have evaluated the effects of lactated Ringer’s solution use on the outcomes of patients suffering from out-of-hospital cardiac arrest. Thus, we examined the association between the use of LR solution before hospital arrival and return of spontaneous circulation (ROSC), 1-month survival, and neurological or physical outcomes at 1 month after the event.  Methods and Findings  We conducted a prospective, non-randomized, observational study using national data from a whole sample of out-of-hospital cardiac arrests between 2005 and 2009 in Japan. We performed a propensity analysis and examined the association between prehospital intravenous loading with lactated Ringer’s solution and short- and long-term survival. The study patients were ≥18 years of age, had an OHCA before arrival of EMS personnel, were treated by EMS personnel, and were then transported to medical institutions.  531,854 out-of-hospital cardiac arrest cases that met the inclusion criteria were analyzed. Among propensity-matched patients, positive or negative associations were observed between prehospital lactated Ringer’s solution and return of spontaneous circulation (ROSC) before hospital arrival and 1-month survival with minimal neurological or physical impairment (ROSC: odds ratios [ORs] (95% CI) = 1.011 (1.001-1.021), 1.264 (1.193-1.339), 1.262 (1.182-1.345), 1.254 (1.166-1.349), 1.239 (1.146-1.339) (p = 0.04 for the first unadjusted model and p = 0.000 for the remaining adjusted models); cerebral performance category (1 or 2): ORs (95% CI) = 0.873 (0.766-0.995), 0.773 (0.609-0.982), 0.764 (0.589-0.992) (ps = 0.04, 0.04, 0.04, respectively, for the adjusted models); and overall performance category (1 or 2): ORs (95% CI) = 0.873 (0.766-0.995), 0.777 (0.611-0.988), 0.746 (0.573-0.971) (ps = 0.04, 0.04, 0.03, respectively, for the adjusted models). Several limitations must be acknowledged. First, data on in-hospital CPR after hospital arrival were not included in analyses. Second, the major limitation was that prehospital LR solution use was not assigned randomly.  Conclusion  Among patients with out-of-hospital cardiac arrest in Japan, prehospital intravenous loading with lactated Ringer solution was independently associated with decreased 1-month survival with minimal neurological impairment but increased ROSC before hospital arrival. However, prehospital lactated Ringer solution use was not associated with 1-month survival. Further study is necessary to verify the present findings. |
| **Introduction** |  |  |
| Background/rationale | 2 | *Explain the scientific background and rationale for the investigation being reported*  Thus far, the outcome of routine IV fluid administration to no fluid administration during CPR in human have not been directly compared. Most human and animal studies of fluid infusion during CPR did not have a control group [11,12], and 2 animal studies showed that normothermic fluid infusion during CPR caused a decrease in CPP [13,14]. In addition to normothermic fluid, hypertonic and chilled fluids have been studied in animal and small human studies without a survival benefit [11,12,15]. Specifically, no studies have evaluated the effects of using prehospital LR solution on outcomes of patients with OHCA. |
| Objectives | 3 | *State specific objectives, including any prespecified hypotheses*  [W]e performed a propensity analysis using national data of all OHCAs between 2005 and 2009 in Japan and examined the association between the use of LR solution before hospital arrival and return of spontaneous circulation (ROSC), 1-month survival, and neurological or physical outcomes at 1 month after the event. |
| **Methods** |  |  |
| Study design | 4 | *Present key elements of study design early in the paper*  This was a prospective observational study using national registry data. |
| Setting | 5 | *Describe the setting, locations, and relevant dates, including periods of recruitment, exposure, follow-up, and data collection*  The emergency medical service (EMS) system in Japan has been explained previously [17,18]. Briefly, EMS was provided through 807 fire stations with municipal government dispatch centers, and a tiered response (#119 for fire and ambulance). As the Japanese guidelines do not allow EMS providers to terminate resuscitation in the field, all patients with OHCA who are treated by EMS personnel are transported to hospitals, excluding those with decapitation, incineration, decomposition, rigor mortis, or dependent cyanosis [19]. An ambulance crew consists of three EMS personnel, including as least one emergency lifesaving technician. They carry a defibrillator which monophasic or biphasic waveform can be selected. Specially trained emergency lifesaving technicians have been permitted to insert an intravenous line with approval from an on-line emergency physician as prehospital emergency care since July 2004 [17, 20]. Since Medical Practitioner’s Law prohibits medical treatments by other than medical doctors in Japan [21], this on-line control is required across the country. Other than inserting an intravenous line, epinephrine administration and advanced airway management also require approval from an on-line emergency physician. The necessity of these advanced life support (ALS) is judged based upon a patient’s condition. The Fire and Disaster Management Agency (FDMA) registers all OHCA cases in a prospective, nationwide, population-based database using the standardized Utstein style template. These data are initially handwritten. Then, EMS personnel in cooperation with the physicians in charge of the patients with OHCA summarize each OHCA case in the standardized Utstein style [19, 22]. Data from the 807 fire stations with dispatch centers in the 47 prefectures are then integrated into a national registry system on the FDMA database server after an electronic data check by FDMA. |
| Participants | 6 | *(a) Give the eligibility criteria, and the sources and methods of selection of participants. Describe methods of follow-up*  The study patients were ≥18 years of age, had an OHCA before arrival of EMS personnel, were treated by EMS personnel, and were then transported to medical institutions. |
|  |  | *(b) For matched studies, give matching criteria and number of exposed and unexposed*  Prehospital LR solution use was not randomly assigned to the patient population. Thus, in order to control for potential confounding and selection biases, we developed a propensity score for LR solution use before hospital arrival for matching rather than using the propensity score as a covariate. Specifically, in the first step, a full non-parsimonious logistic regression model with prehospital LR solution use as a dependent variable, which included as independent variables every variable except for the three endpoint variables (i.e., 1-month survival, and CPC and OPC scores) in Table 1 (i.e., 67 variables including four dummy variables for “cases by year” and 46 dummy variables for the 47 prefectures in Japan) was fitted. A propensity score for LR solution use before hospital arrival was calculated from the logistic regression equation for each patient. This propensity score represented the probability of prehospital LR solution use. In the second step, based on the propensity score, cases that used prehospital LR solution were matched to unique control patients who did not use prehospital LR solution. |
| Variables | 7 | *Clearly define all outcomes, exposures, predictors, potential confounders, and effect modifiers. Give diagnostic criteria, if applicable*  Endpoints were ROSC before hospital arrival; survival at 1 month after cardiac arrest; survival with minimal neurological impairment, defined as CPC category 1 or 2; and survival with minimal neurological disability, defined as OPC category 1 or 2. Exposure was the use of lactated Ringer’s solution. Covariates were cases by year, age, sex, bystander eyewitness, relationship between bystander and patient, bystander chest compression, bystander rescue breathing, use of public-access AED by bystander, first documented rhythm, origin of OHCA, time from call to arrival at the scene, and time from call to arrival at hospital. |
| Data sources/measurement | 8* | *For each variable of interest, give sources of data and details of methods of assessment (measurement). Describe comparability of assessment methods if there is more than one group*  The Fire and Disaster Management Agency (FDMA) registers all OHCA cases in a prospective, nationwide, population-based database using the standardized Utstein style template. These data are initially handwritten. Then, EMS personnel in cooperation with the physicians in charge of the patients with OHCA summarize each OHCA case in the standardized Utstein style. Data from the 807 fire stations with dispatch centers in the 47 prefectures are then integrated into a national registry system on the FDMA database server after an electronic data check by FDMA. |
| Bias | 9 | *Describe any efforts to address potential sources of bias*  Prehospital LR solution use was not randomly assigned to the patient population. Thus, in order to control for potential confounding and selection biases, we developed a propensity score for LR solution use before hospital arrival for matching rather than using the propensity score as a covariate. |
| Study size | 10 | *Explain how the study size was arrived at*  With an actual ROSC, 1-month survival, CPC category 1 or 2 after the event, OPC category 1 or 2 after the event rates of 6.29%, 4.25%, 1.59%, and 1.58% in the prehospital LR solution group and 5.22%, 4.07%, 1.79% and 1.79% in the prehospital no-LR solution group of propensity-matched subjects, 76,293 samples for each group provided acceptable power levels (i.e. > 0.80) with a type I error of 5% or 1%. |
| Quantitative variables | 11 | *Explain how quantitative variables were handled in the analyses. If applicable,*  *describe which groupings were chosen and why*  Neurological outcomes 1 month after the event were evaluated using the five categories of the Cerebral Performance Category (CPC) Scale (1: good cerebral performance; 2: moderate cerebral disability; 3: severe cerebral disability; 4: coma or vegetative state; 5: death). Physical status 1 month after the event was evaluated using the five categories of the Overall Performance Category (OPC) Scale (1: no or mild neurological disability; 2: moderate neurological disability; 3: severe neurological disability; 4: coma or vegetative state; 5: death). Then both CPC and OPC were categorized into two groups: 1 or 2 vs. 3 to 5. |
| Statistical methods | 12 | *(a) Describe all statistical methods, including those used to control for confounding*  Of the data of patients who had OHCAs between January 1, 2005, and December 31, 2009, in Japan and who were entered into the national registry (n = 547,218), data that met the criteria concerning patient age and time course were analyzed (n = 531,854) (Fig. 1).  Prehospital LR solution use was not randomly assigned to the patient population. Thus, in order to control for potential confounding and selection biases, we developed a propensity score for LR solution use before hospital arrival for matching rather than using the propensity score as a covariate. Specifically, in the first step, a full non-parsimonious logistic regression model with prehospital LR solution use as a dependent variable, which included as independent variables every variable except for the three endpoint variables (i.e., 1-month survival, and CPC and OPC scores) in Table 1 (i.e., 67 variables including four dummy variables for “cases by year” and 46 dummy variables for the 47 prefectures in Japan) was fitted. A propensity score for LR solution use before hospital arrival was calculated from the logistic regression equation for each patient. This propensity score represented the probability of prehospital LR solution use. In the second step, based on the propensity score, cases that used prehospital LR solution were matched to unique control patients who did not use prehospital LR solution. The matching algorithms was greedy match which is frequently used to match cases to controls in observational studies, and once a match is made, the match is not reconsidered. Using data for all cases, three unconditional multiple logistic regression models were fit using one of the endpoint variables in Table 1 as a dependent variable. Using the propensity-matched patient data, conditional multiple logistic regression models were fit using one of the endpoint variables in Table 3 as a dependent variable. Specifically, starting with an unadjusted model, we adjusted for the effects of (1) propensity, (2) propensity and significant variables in propensity-matched sample in Table 3, (3) propensity, significant variables in propensity-matched sample in Table 3, and variables shown to be a factor of resuscitation outcome, and (4) propensity and all study variables in Table 1. In total, five models were fit. With an actual ROSC, 1-month survival, CPC category 1 or 2 after the event, OPC category 1 or 2 after the event rates of 8.79%, 4.43%, 1.51%, and 1.52% in the prehospital LR solution group and 5.95%, 5.01%, 2.54% and 2.51% in the prehospital no-LR solution group of total subjects (Table 1), 109,140 samples for each group provided the power levels summarized in Table 4 with a type I error of 5% or 1%. With an actual ROSC, 1-month survival, CPC category 1 or 2 after the event, OPC category 1 or 2 after the event rates of 6.29%, 4.25%, 1.59%, and 1.58% in the prehospital LR solution group and 5.22%, 4.07%, 1.79% and 1.79% in the prehospital no-LR solution group of propensity-matched subjects (Table 3), 76,293 samples for each group provided the power levels summarized in Table 4 with a type I error of 5% or 1%. The two-sided significance level for all tests was P < 0.05. All analyses were performed using SAS version 8.2 software (SAS Institute, Cary, NC, USA). |
|  |  | *(b) Describe any methods used to examine subgroups and interactions*  Using the propensity-matched patient data, conditional multiple logistic regression models were fit using one of the endpoint variables in Table 3 as a dependent variable. Specifically, starting with an unadjusted model, we adjusted for the effects of (1) propensity, (2) propensity and significant variables in propensity-matched sample in Table 3, (3) propensity, significant variables in propensity-matched sample in Table 3, and variables shown to be a factor of resuscitation outcome, and (4) propensity and all study variables in Table 1. In total, five models were fit. |
|  |  | *(c) Explain how missing data were addressed*  Of cases with missing values for study variables in Figure 1 (n= 4,272), the number of cases with missing values for age was 62. Age distribution was compared between the remaining cases with missing values for study variables except for age (n= 4,210). |
|  |  | *(d) If applicable, explain how loss to follow-up was addressed*  Not applicable. |
|  |  | *(e) Describe any sensitivity analyses*  According to the resuscitation guidelines, intravenous solution is administered to patients with ROSC to improve hemodynamic stability after hospital arrival [36]. In matched subsets of patients who had ROSC prior to hospital arrival, the opportunity to receive intravenous LR solution would be equal in OHCA patients who received prehospital LR solution and those who did not. Thus, the pure effect of prehospital LR solution use could be determined. The number of 1-month survivors in the LR solution and no-LR solution groups were 1876 (43.30%) and 1721 (46.83%), respectively (P= 0.001). The numbers of patients in CPC categories 1 or 2 in the LR solution and no-LR solution groups were 937 (21.57%) and 1042 (28.35%), respectively (P < 0.0001). The numbers of patients in OPC categories 1 or 2 in the LR solution and no-LR solution groups were 933 (21.48%) and 1040 (28.30%), respectively (P < 0.0001). Additionally, we compared time from the call to hospital arrival in the LR solution with that in the no-LR solution group, and the times were 36.93 ± 14.24 min and 40.43 ± 25.91 min, respectively (P < 0.0001). Although the LR solution group was transported more quickly to the emergency department, and, thus, definitive care could be performed more rapidly, decreased long-term effect was observed. |
| **Results** |  |  |
| Participants | 13* | *(a) Report numbers of individuals at each stage of study—eg numbers potentially*  *eligible, examined for eligibility, confirmed eligible, included in the study,*  *completing follow-up, and analysed*  During the 5 years of the study, 531,854 patients with OHCA met the inclusion criteria (Fig. 1, Table 1). Of cases with missing values for study variables in Figure 1 (n= 4,272), the number of cases with missing values for age was 62. Age distribution was compared between the remaining cases with missing values for study variables except for age (n= 4,210) and total analyzed cases (n= 531,854). |
|  |  | *(b) Give reasons for non-participation at each stage*  Not applicable. |
|  |  | *(c) Consider use of a flow diagram*  Figure 1. Out-of-hospital cardiac arrest (OHCA) cases between 2005 and 2009 which were used for analyses. |
| Descriptive data | 14* | *(a) Give characteristics of study participants (eg demographic, clinical, social) and*  *information on exposures and potential confounders*  A significant difference was observed between the LR and No-LR groups with respect to all variables in Table 1. Specifically, patients who received LR solution were more likely to be intubated (74.23% vs 35.73%). One possible reason for this finding is that EMS staffs who could insert an intravenous line tend to have higher clinical skills and experiences, leading to more prehospital end-tracheal intubation. Patients with cardiac origin were more likely to receive LR solution than patients with non-cardiac origin (21.58% vs 18.93%; χ2= 266.56, p= 0.000). Among patients with ROSC, time from the call to hospital arrival in the LR solution and the no-LR solution groups were 36.93 ± 14.24 min and 40.43 ± 25.91 min, respectively (P < 0.0001). |
|  |  | *(b) Indicate number of participants with missing data for each variable of interest*  Of cases with missing values for study variables in Figure 1 (n= 4,272), the number of cases with missing values for age was 62. Age distribution was compared between the remaining cases with missing values for study variables except for age (n= 4,210) and total analyzed cases (n= 531,854). |
|  |  | *(c) Summarise follow-up time (eg, average and total amount)*  (Each case was followed up at 1-month after OHCA.) |
| Outcome data | 15* | *Report numbers of outcome events or summary measures over time*  Table 2 summarizes patient outcomes based on prehospital intravenous loading with LR and four types of outcome measures among all subjects. A significant and negative association was detected between intravenous loading with LR solution before hospital arrival (OR=1.521, 95% CI, 1.484-1.559, p=0.000 in “unadjusted”; OR=0.951, 95% CI, 0.921-0.983, p=0.003 in “adjusted for selected variables”; OR=1.194, 95% CI, 1.153-1.237, p=0.000 in “adjusted for all covariates”) and CPC (OR=0.880, 95% CI, 0.852-0.909, p=0.000 in “unadjusted”; OR=0.796, 95% CI, 0.766-0.828, p=0.000 in “adjusted for selected variables”; OR=0.986, 95% CI, 0.946-1.029, p=0.52 in “adjusted for all covariates”) and OPC (OR=0.598, 95% CI, 0.567-0.630, p=0.000 in “unadjusted”; OR=0.531, 95% CI, 0.499-0.565, p=0.000 in “adjusted for selected variables”; OR=0.782, 95% CI, 0.732-0.836, p=0.000 in “adjusted for all covariates”) in the three types of models. |
| Main results | 16 | *(a) Give unadjusted estimates and, if applicable, confounder-adjusted estimates and*  *their precision (eg, 95% confidence interval). Make clear which confounders were*  *adjusted for and why they were included*  Prehospital intravenous loading with LR in all OHCA cases and patient outcome  Table 2 summarizes patient outcomes based on prehospital intravenous loading with LR and four types of outcome measures among all subjects. A significant and negative association was detected between intravenous loading with LR solution before hospital arrival (OR=1.521, 95% CI, 1.484-1.559, p=0.000 in “unadjusted”; OR=0.951, 95% CI, 0.921-0.983, p=0.003 in “adjusted for selected variables”; OR=1.194, 95% CI, 1.153-1.237, p=0.000 in “adjusted for all covariates”) and CPC (OR=0.880, 95% CI, 0.852-0.909, p=0.000 in “unadjusted”; OR=0.796, 95% CI, 0.766-0.828, p=0.000 in “adjusted for selected variables”; OR=0.986, 95% CI, 0.946-1.029, p=0.52 in “adjusted for all covariates”) and OPC (OR=0.598, 95% CI, 0.567-0.630, p=0.000 in “unadjusted”; OR=0.531, 95% CI, 0.499-0.565, p=0.000 in “adjusted for selected variables”; OR=0.782, 95% CI, 0.732-0.836, p=0.000 in “adjusted for all covariates”) in the three types of models.  Prehospital LR solution loading and outcome in propensity-matched patients  The propensity scores ranged from 0.003 to 0.997, indicating that the probability of intravenous loading with LR before hospital arrival would be between 0.003 and 0.997. This model yielded a c statistic of 0.85, indicating a strong ability to differentiate between intravenous loading with LR before hospital arrival and other cases. In total, 76,293 prehospital intravenous loading with LR cases were matched to 76,293 other unique cases (Table 3). No significant differences were detected between the “LR use” and “No-LR use” groups with respect to independent variables except for “cases by year” (p = 0.002), “bystander eyewitness” (p = 0.03), “emergency life-saving technician in ambulance” (p = 0.007), “time from call to hospital arrival” (p = 0.001), and “epinephrine use” (p = 0.000).  Table 5 summarizes survival outcomes based on prehospital intravenous loading with LR solution among propensity-matched patients. A significant and positive association was observed between prehospital intravenous loading with LR solution and the outcome measures in the five analysis models (OR=1.011, 95% CI, 1.001-1.021, p=0.04 in “unadjusted”; OR=1.264, 95% CI, 1.193-1.339, p=0.000 in “adjusted for propensity”; OR=1.262, 95% CI, 1.182-1.345, p=0.000 in “adjusted for propensity and significant variables in Table 3” ; OR=1.254, 95% CI, 1.166-1.349, p=0.000 in “adjusted for propensity, significant variables in Table 3, and selected variables”; OR=1.239, 95% CI, 1.146-1.339, p=0.000 in “adjusted for propensity and all covariates”) for ROSC. As for survival with minimal neurological impairment, significant negative associations were observed between prehospital intravenous LR loading and CPC category 1 or 2 and OPC category 1 or 2 in the models after adjusting for propensity and significant variables in Table 3 (OR=0.873, 95% CI = 0.766-0.995, p = 0.04; and OR=0.873, 95% CI=0.766-0.995, p=0.04, respectively), for propensity, significant variables in Table 3, and selected variables (OR=0.773, 95% CI = 0.609-0.982, p = 0.04; and OR=0.777, 95% CI=0.611-0.988, p=0.04, respectively), and for propensity and all covariates (OR=0.764, 95% CI = 0.589-0.992, p = 0.04; and OR=0.746, 95% CI=0.573-0.971, p=0.03, respectively). |
|  |  | *(b) Report category boundaries when continuous variables were categorized*  Not applicable. |
|  |  | *(c) If relevant, consider translating estimates of relative risk into absolute risk for a*  *meaningful time period*  Not applicable. |
| Other analyses | 17 | *Report other analyses done—eg analyses of subgroups and interactions, and sensitivity analyses*  We conducted a sensitivity analysis. According to the resuscitation guidelines, intravenous solution is administered to patients with ROSC to improve hemodynamic stability after hospital arrival. In matched subsets of patients who had ROSC prior to hospital arrival, the opportunity to receive intravenous LR solution would be equal in OHCA patients who received prehospital LR solution and those who did not. Thus, the pure effect of prehospital LR solution use could be determined. The number of 1-month survivors in the LR solution and no-LR solution groups were 1876 (43.30%) and 1721 (46.83%), respectively (P= 0.001). The numbers of patients in CPC categories 1 or 2 in the LR solution and no-LR solution groups were 937 (21.57%) and 1042 (28.35%), respectively (P < 0.0001). The numbers of patients in OPC categories 1 or 2 in the LR solution and no-LR solution groups were 933 (21.48%) and 1040 (28.30%), respectively (P < 0.0001). Additionally, we compared time from the call to hospital arrival in the LR solution with that in the no-LR solution group, and the times were 36.93 ± 14.24 min and 40.43 ± 25.91 min, respectively (P < 0.0001). Although the LR solution group was transported more quickly to the emergency department, and, thus, definitive care could be performed more rapidly, decreased long-term effect was observed. In summary, the sensitivity analysis showed that prehospital LR solution use might be related to decreased 1-month survival with minimal neurological impairment. |
| **Discussion** |  |  |
| Key results | 18 | *Summarise key results with reference to study objectives*  We revealed that prehospital intravenous loading with LR solution was independently associated with decreased 1-month survival with minimal neurological impairment (i.e., CPC category 1 or 2, OPC category 1 or 2) and increased ROSC before hospital arrival (Tables 3 and 5 ). |
| Limitations | 19 | *Discuss limitations of the study, taking into account sources of potential bias or imprecision. Discuss both direction and magnitude of any potential bias*  Several limitations and caveats to our study must be acknowledged. First, data on in-hospital CPR after hospital arrival were not included in analyses. It is possible that our findings may have been due to a difference in in-hospital resuscitation, such as hypothermia and mechanical chest compression devices among the LR solution and no-LR solution groups. Although the quality of in-hospital resuscitation might influence 1-month survival, we could not control for the effects of such factors. Second, the major limitation was that prehospital LR solution use was not assigned randomly. We performed a propensity analysis and made a rigorous adjustment for selection bias and confounding factors, which would be expected with a standard multivariate analysis. Nevertheless, we acknowledge that observational studies can only partially control and adjust for factors actually measured, whereas randomized allocation can control both known and unknown confounding factors and avoid introducing bias. |
| Interpretation | 20 | *Give a cautious overall interpretation of results considering objectives, limitations, multiplicity of analyses, results from similar studies, and other relevant evidence*  In summary, we revealed that prehospital intravenous loading with LR solution was independently associated with decreased 1-month survival with minimal neurological or physical impairment and increased ROSC before hospital arrival. |
| Generalisability | 21 | *Discuss the generalisability (external validity) of the study results*  Although there is a difference between the data analyzed in the previous and the present studies (i.e., Utstein data for 2005–2008 vs. 2005–2009), the effects of epinephrine on the outcome variables seems to be greater than that of LR solution. Specifically, the ORs of prehospital epinephrine use were between 1.91 and 2.51 for return of spontaneous circulation (ROSC) before hospital arrival, whereas the ORs of prehospital LR solution use were between 1.01 and 1.26 for ROSC before hospital arrival (Table 5). The ORs of prehospital epinephrine use were between 0.21 and 0.41 and between 0.23 and 0.43 for CPC (1, 2) and OPC (1, 2), respectively, whereas those of prehospital LR solution use were between 0.76 and 0.87 and between 0.75 and 0.87, respectively (Table 5). In this study, after propensity matching, a difference between the LR and No-LR groups remained with respect to epinephrine use, with the LR group using epinephrine more frequently than the No-LR group did (1.70% vs. 1.25%, p = 0.00) (Table 3). This might be why the models that did not control for the effects of prehospital epinephrine use reported no significant association between prehospital LR solution use and CPC (1, 2) or OPC (1, 2) (Table 5). |
| **Other information** |  |  |
| Funding | 22 | *Give the source of funding and the role of the funders for the present study and, if applicable, for the original study on which the present article is based*  No specific funding was received for this study. No funding was required or used during the writing of this paper, none of the authors were salaried during the time of writing and no funders had any role in the study design, data collection, analysis, decision to publish or preparation of the manuscript. |
| **Give information separately for exposed and unexposed groups.* | | |
